# FITBAR: a web tool for the robust prediction of prokaryotic regulons

**DOI:** 10.1186/1471-2105-11-554

**Published:** 2010-11-11

**Authors:** Jacques Oberto

**Affiliations:** 1 Université Paris-Sud 11, CNRS, UMR8621, Institut de Génétique et Microbiologie, 91405 Orsay, France

## Abstract

**Background:**

The binding of regulatory proteins to their specific DNA targets determines the accurate expression of the neighboring genes. The *in silico *prediction of new binding sites in completely sequenced genomes is a key aspect in the deeper understanding of gene regulatory networks. Several algorithms have been described to discriminate against false-positives in the prediction of new binding targets; however none of them has been implemented so far to assist the detection of binding sites at the genomic scale.

**Results:**

FITBAR (Fast Investigation Tool for Bacterial and Archaeal Regulons) is a web service designed to identify new protein binding sites on fully sequenced prokaryotic genomes. This tool consists in a workbench where the significance of the predictions can be compared using different statistical methods, a feature not found in existing resources. The Local Markov Model and the Compound Importance Sampling algorithms have been implemented to compute the P-value of newly discovered binding sites. In addition, FITBAR provides two optimized genomic scanning algorithms using either log-odds or entropy-weighted position-specific scoring matrices. Other significant features include the production of a detailed genomic context map for each detected binding site and the export of the search results in spreadsheet and portable document formats. FITBAR discovery of a high affinity *Escherichia coli *NagC binding site was validated experimentally *in vitro *as well as *in vivo *and published.

**Conclusions:**

FITBAR was developed in order to allow fast, accurate and statistically robust predictions of prokaryotic regulons. This feature constitutes the main advantage of this web tool over other matrix search programs and does not impair its performance. The web service is available at http://archaea.u-psud.fr/fitbar.

## Background

In every living organism, the binding of regulatory proteins to their specific DNA targets accounts for the accurate transcription modulation and expression of the neighboring genes. The prediction, *in silico*, of new transcription factor binding sites (TFBSs) is a key aspect of the deeper understanding of gene regulation. The discovery of regulons, sets of functionally related and co-regulated genes scattered throughout the genome, is of great importance for the geneticist. However, the exponentially growing number of fully sequenced genomes, especially prokaryotic, has turned the prediction of regulons into a daunting task. Several reviews compare the algorithms that have been developed to address the identification of TFBSs [1-5]. These programs can be subdivided into two main classes. In the first class, DNA binding sites are predicted in a limited amount of short sequences where a particular regulation is known to occur but without prior knowledge on the binding site sequence itself. These *de novo *search algorithms detect over-represented or non-random information pertaining to binding sites by the means of probabilistic approaches such as Gibbs sampling, hidden Markov models and their variations. In the second category of programs, binding sites can be predicted on DNA sequences of any length. The only prerequisite in this case is a list of known binding sites sharing the same biological properties, determined experimentally. These properly aligned sequences define the position-specific scoring matrix (PSSM), a flexible representation of the binding motif [6]. PSSMs have been widely used to detect motifs in DNA or protein sequences [7]. Unlike probabilistic *de novo *approaches, PSSM search programs are not limited by the size or the number of the DNA sequences and are therefore particularly well suited to scan entire genomes and predict regulons. The program ScanAce constituted the initial implementation of a PSSM DNA search tool; it involved the manual handling of DNA sequence files and required program execution on the local system shell exclusively [8]. Novel scanning algorithms such as QPMEME [9] or OPENFILL/SCANGEN [10] based on the estimate sequence-specific binding energy of a given transcription factor have been reported. However, these computing techniques do not seem to solve the problem of the false negatives [10]. Furthermore, QPMEME fails to find a solution on datasets containing many low affinity sequences [11]. More recently, the availability of a large and growing number of completely sequenced prokaryotic genomes triggered a regain in interest for PSSM searches. These genomic databases permitted the development of web services such as MAST [12], RSA Tools [13], PredictRegulon [14], PRODORIC Virtual Footprint [15] and RegPredict [16] to grant easier access to genome-wide regulon prediction. Unfortunately, the results of existing PSSM genomic scanning programs rely on the choice of an arbitrary threshold value. A low threshold may detect a large number of false positive sites whereas a high threshold may fail to produce any meaningful result. The MAST web service can produce TFBS P-values but only analyzes intergenic regions; furthermore, its results are not computed in real time. Despite the fact that a considerable progress has been made in assessing the statistical significance in biological sequence analysis [17], the interactive prediction of regulons using probabilistic methods remains a computationally intensive task and appropriate computer programs are not available. To address this problem, I have developed FITBAR (Fast Investigation Tool for Bacterial and Archaeal Regulons), a real-time PSSM scanning web tool for completely sequenced prokaryotic genomes. FITBAR is designed as a high-performance workbench providing two algorithms for the detection of new binding sites in combination with two methods to calculate their P-values. This web service aims to assist the experimentalist with the discovery and characterization of new prokaryotic regulons.

## Implementation

### Web service and database implementation

The FITBAR web service is developed in the C# language and ASP.NET web scripting language. The application is deployed on a server equipped with two quad-core AMD Opteron 8378 processors clocked at 2.4 Ghz and 8 GB of RAM. The operating system is Windows Server 2008 RC2. The service is freely accessible from any operating system/internet browser combination at the URL http://archaea.u-psud.fr/fitbar. All computations and predictions are executed interactively in real time. The server stores over 200 consensus prokaryotic binding sites matrices collected from Harvard University http://arep.med.harvard.edu/ecoli_matrices/ and from RegTransBase http://regtransbase.lbl.gov/cgi-bin/regtransbase. Alternatively, user-defined matrices in Fasta or raw format can be submitted as well. FITBAR genomic databases are stored on the server and provide access to the publicly available complete genomes of Bacteria and Archaea. An accessory program enables the daily automated update of the database from the repository of the National Center for Biotechnology Information (NCBI) ftp://ftp.ncbi.nih.gov/genbank/genomes/Bacteria using the FTP protocol. Newly sequenced genomes are therefore available within 24 hrs of their public release. Genomes are downloaded in the GenBank format and parsed to extract the information relevant to FITBAR. These flat file genomic databases are shared with the BAGET web server http://archaea.u-psud.fr/bin/baget.dll and were described previously [18]. In addition, for every represented prokaryotic chromosome, the database contains a table of cumulative mono-, di-, tri- and tetranucleotide frequencies used to generate the Markov models. This table is computed once, each time a new chromosome is added to the database. The generation of reports in Portable Document Format (PDF) is achieved using the open-source PDFsharp library http://www.pdfsharp.net/

### PSSM scanning algorithms

Two methods have been described to score candidate sequences for their similarities to known binding sites using position specific scoring matrices. The nucleotide distribution frequencies at each position are computed from an aligned series of biologically defined binding sites. These frequencies can then be transformed using either the log-odds [19] or the entropy-weighted [20] algorithms to generate the PSSM. Query sites are then matched against the PSSMs by summing up the score at each corresponding position. In this work, both log-odds and entropy-weighted search algorithms have been implemented as optimized multithreaded routines in order to scan both DNA strands simultaneously and to take advantage of multi-core processors. For compatibility purposes, the scores obtained with the two scanning algorithms are normalized to 1.0 according to the best theoretical binding site deduced from the PSSM.

### Compound Importance Sampling

The methodology to calculate P-values using the compound importance sampling has been described [21]. This variance-reduction technique of Monte Carlo estimators can be used as an efficient alternative to naïve direct simulation [17]. Briefly, each genomic query requires the generation of 10 compounds containing respectively 9986, 7732, 5987, 4636, 3590, 2780, 2153, 1667, 1291 and 1000 samples. The samples consist of Markov chains generated using the null model and mixed with samples from the consensus sites, in a mixing ratio that varies linearly from 0 to 1. Each sample is scored against the consensus motif and the results are compiled to generate a distribution. The cumulated frequencies of this distribution allow the calculation of the P-value, for each score. Since the compounds contain samples from the user-specified consensus sites, they need to be computed at query time. The background model is constituted by third-order Markov chains generated at query time using the pre-calculated chromosome-specific mono- to tetranucleotide frequencies from the database. The Bonferroni correction for multiple comparisons was not be used in this implementation due the large number of repetitions involved in the scanning of entire genomes. The CIS algorithm was implemented in FITBAR according to the description in the original article and additional information (T. Kaplan, pers. comm.).

### Local Markov Model

**Figure 1:**
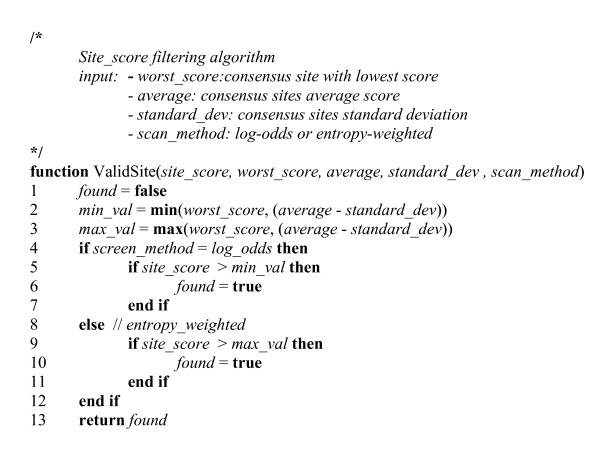
**Algorithm for the Local Markov Method filtering heuristics formulated in pseudocode**.

The Local Markov Model uses an efficient algorithm based on probability-generating functions to compute the P-value of candidate binding sites [22]. Briefly, the candidate binding site sequences are first scored by PSSMs then submitted to filtering. For this implementation, a different filtering heuristics was developed (Figure 1). It takes into account the distribution of predicted TFBS score values which is more dispersed for the log-odds than for the entropy-weighted method. The same behavior was observed for all PSSM tested (data not shown). The P-value of the pre-selected sites are computed against a null model based on the local genomic context. This null model or background distribution is constituted by a second-order Markov chain computed on the basis of a 1000 nucleotide segment surrounding the predicted binding site, excluding the actual binding sequence. This P-value algorithm is limited to PSSMs with an informational content > 12 bit. The LMM algorithm in FITBAR consists of a C# implementation based on the original C++ source code [22].

### Sequence logo

A PSSM can be represented under the form of a sequence logo pictogram showing the consensus sequence, the relative frequency of bases and the informational content (measured in bit) [23]. Sequence logos were originally developed with the PostScript description language and fonts. In the present implementation, the processing overhead imposed by the PostScript language was eliminated by using a more efficient OpenType font rendering. This implementation includes a small sample correction to avoid underestimation of the entropy for query datasets composed of a limited amount of sequences [24].

## Results and Discussion

In spite of the number of existing software tools to identify specific DNA binding sites for regulatory proteins [25,26], the continuous development of new programs illustrate the fact that the optimal TFBS prediction system is not available yet. The prediction of regulons remains a non-trivial and time consuming task for the experimentalist, especially for the analysis of the large and growing number of completely sequenced prokaryotic genomes. A common limitation of the existing PSSM search programs reside in the selection mechanism for the newly detected binding sites. It is achieved mainly by discarding sites presenting a PSSM similarity score below an arbitrary threshold value. With the naive assumption that prediction errors accumulate proportionally to the length of the scanned DNA sequence, the validity of PSSM searches over an entire genome is questionable in the absence of a proper statistical analysis. On the other hand, the elimination of false positives by classical statistical methods is inadequate for real time analysis. For an average sized prokaryotic chromosome, this correction would exceed, by two to three orders of magnitude, the computing time required for the initial genome scan [21]. The FITBAR web service was developed to bring a solution to this problem by providing an interactive and statistically significant prediction of DNA binding sites at the genomic scale.

### General features

**Figure 2:**
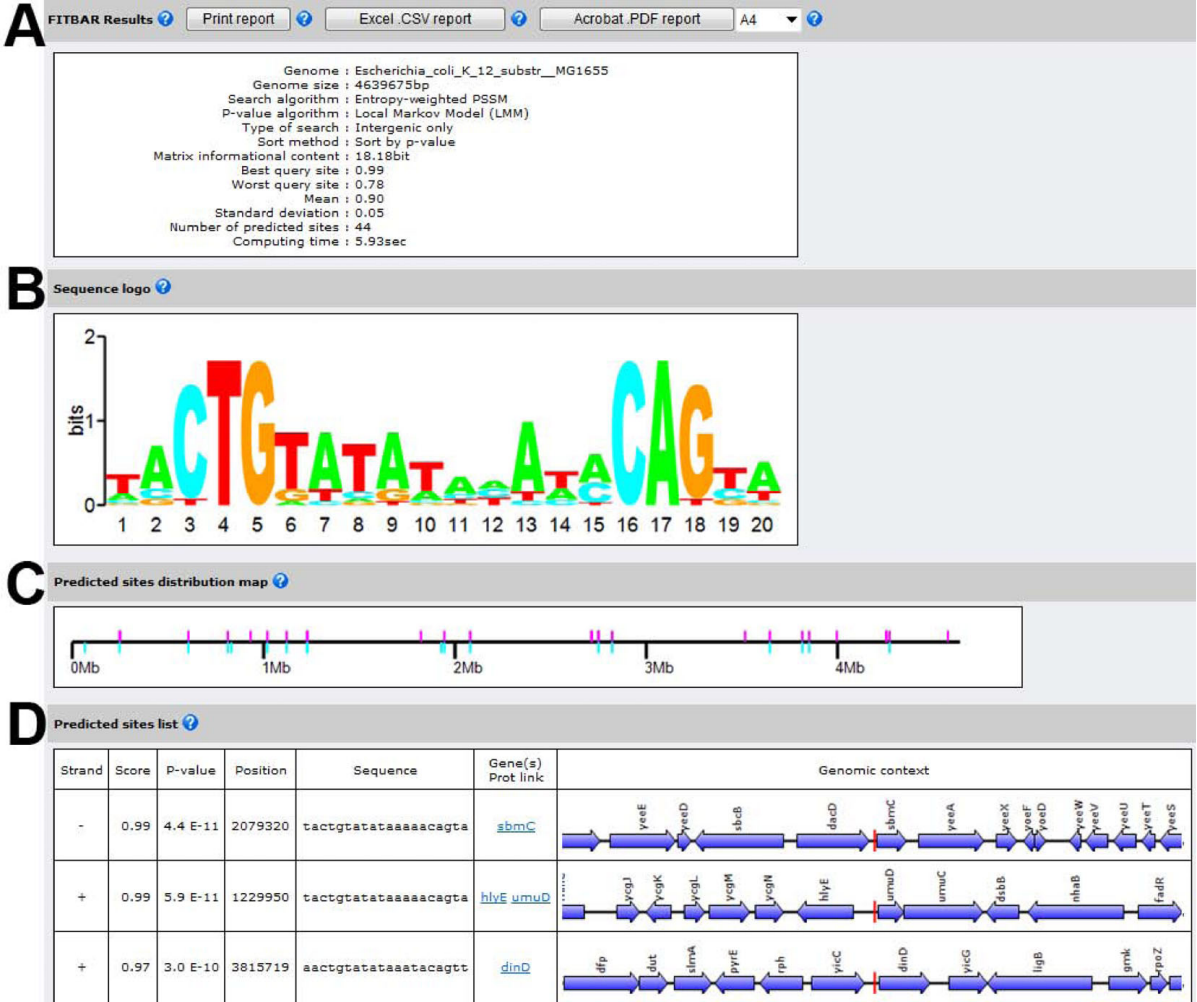
**FITBAR report for the *Escherichia coli *LexA repressor binding sites**. (A) The site statistics panel displays numerical information on the selected chromosome and consensus binding site. (B) The sequence logo permits to evaluate visually the information content of the consensus motif. (C) The site distribution panel indicates the distribution of the new sites on both DNA strands of the chromosome. (D) The binding site list shows FITBAR results for each newly discovered site such as position, sequence, orientation, score, P-value, gene product information and detailed genomic context map of 10 KB (partial view).

The FITBAR web tool was developed in C# and the choice of this particular language was motivated by its performance over other commonly used programming languages. Memory usage and the reading of large sequence files is more efficient in C# than Java; the speed of execution is nearly as fast as C and C++ and 6 × faster than Perl and Python [27]. The web service consists of dynamic web pages compatible with all current internet browsers and operating systems. FITBAR relies on the same genomic databases as the BAGET web tool [18]. The data files are stored locally to increase performance and undergo a daily automated update from the National Center for Biotechnology Information (NCBI) repository (see Implementation). In order to select individual chromosomes, the user is provided with a list of bacterial and archaeal species names. Organism names have been appended the C1, C2, etc, suffixes when they harbor multiple replicons. The only external data required by FITBAR consist of an aligned series of known binding sites which can be copy-pasted directly in the appropriate text area. Alternatively, binding sites can be selected from the local database providing over 200 known prokaryotic matrices. FITBAR will generate a PSSM consensus and search a selected chromosome for additional sites using the log-odds or entropy-weighted algorithms. Queries can be conducted on entire chromosomes or restricted to intergenic regions. The statistical significance of potential binding sites can be assessed either by the Local Markov Model or Compound Importance Sampling algorithms (see next section). If the query is successful, FITBAR will provide a graphically-rich report composed of four parts. (1) The first panel details chromosome and query sites statistics in addition to the user-selected scanning and P-value methods (Figure 2A). (2) A sequence logo permits a visual quantization of the informational content at every nucleotide of the query PSSM [23] (see Implementation). (Figure 2B). (3) A map drawn to scale permits to evaluate rapidly the predicted binding sites distribution on the entire chromosome (Figure 2C). (4) The binding site list details, for every predicted target, its chromosomal position, orientation, score, P-value, DNA sequence and detailed genomic context graphical map over 10 KB; in addition it provides, for each potential regulated gene, a link to the encoded function at the NCBI database (Figure 2D). FITBAR search results can be either printed or exported in Excel. CSV format for further elaboration and in portable document format for storage or device-independent high-resolution printing.

### Statistical significance of the newly predicted sites: P-value algorithms

The principal objective of FITBAR is to predict statistical significant TFBSs. This significance is commonly assessed by computing a P-value which measures the probability of its stochastic occurrence. P-values can be calculated either with analytic expressions describing the score distributions or alternatively by simulation; several efficient algorithms have been proposed for both approaches [17]. For the present work, two P-value algorithms were retained: the Compound Importance Sampling (CIS) [21] and Local Markov Model (LMM) [22] (see Implementation for a description). The first criteria that motivated this choice was the background model used by the algorithms. Both rely on Markov (of order m ≥ 2) models for the null distribution which have been shown to represent accurately biological DNA sequences [22,28]. This observation is particularly relevant to prokaryotic genomes where the sequence composition varies considerably. It has been reported that the GC-content ranges from 23.7% in *Mycoplasma bovoculi *to 69.5% in *Pseudomonas pseudomallei *[29]. The second criteria was the speed of execution since fast algorithms would be better suited for an interactive web service. Finally, it seemed worthwhile to compare the efficiency of analytical and simulation methods. The two algorithms were adapted to the FITBAR web service as follows. The implementation of CIS was straightforward: it is executed once per genome/query and a unique P-value is assigned to each possible PSSM score before the actual genome scan. FITBAR therefore evaluates the PSSM significance at each genomic position and retains the candidate sites below a cutoff P-value (see next section). The LLM algorithm is based on the local genomic context and requires therefore the calculation of a specific background distribution for each potential site. In the original description, this computation-intensive task is restricted to the top 0.1% candidate sites based on their PSSM similarity scores [22]. This filtering method is impractical for complete genomes as it could require an excess of 10^4 ^individual background calculations for large chromosomes. FITBAR uses a different LLM sorting heuristics as shown in Figure 1. It is based on the query binding site statistics and on the difference in score distribution between the entropy-weighted and log-odds screening algorithms (see next section). A further restriction has been imposed on degenerated PSSMs with a sequence logo informational content < 12 bit which are not considered for the LMM algorithm. The accuracy of the predictions is also assessed in FITBAR by the calculation of the Receiver Operating Characteristic Curve (ROC) which visualizes the components of the false discovery rate. More precisely, the Area Under the Receiver Operating Characteristic Curve (AUROC), a common summary statistic proportional to the quality of a predictor in a binary classification task [30], is provided for each PSSM search.

### Benchmarking and experimental validation

The principal aim of FITBAR is to predict and rank TFBSs by their P-value. This feature is not found in other PSSM scanning programs and it was therefore important to contribute to its development. Since the two selected P-value algorithms were developed originally to analyze short sequences, their performance was tested extensively for genomic scanning and one of these experiments is detailed below. Necessary adjustments were performed to allow the prediction of statically significant TFBS on complete chromosomes.

Benchmarking tests were conducted as follows to illustrate the performance and functionality of FITBAR and to compare it to other available genomic PSSM scanning tools. The *Escherichia coli *K12 MG1655 genome was screened for potential binding sites for the transcriptional regulator NagC involved in *N*-acetylglucosamine metabolism. The query sites are shown in Table 1 and consisted of known NagC operator sequences compiled from [31] and J. Plumbridge (pers. comm.).

**Table 1:** List of *E. coli *NagC binding sites used for benchmarking

Binding sites	* E. coli *genes
CTTATTTTATCATTCAAAAAATC	*nagB*
TTTAATTTGCGATACGAATTAAA	*nagE*
CTTAATTATCTTCGCGAATTATT	*chbB *distal
GATATTTTACCTTTCGAAATTTC	*man *distal
CATAATTCTCATCATGAAATATG	*fimB2*
GTTTATTCATTGATCGAAATAAG	*glmU *distal
TGCAATTCGTGTCACAAAATATG	*fimB1*
CTTATTTCTCTTCGTAAAATTAC	*ydeN1 *proximal
GTTGTTTATCGGCGAGAAATTAC	*ydeN2 *middle
GATAATTCGCGTCGCGAAAAATA	*ybfM *proximal

**Figure 3:**
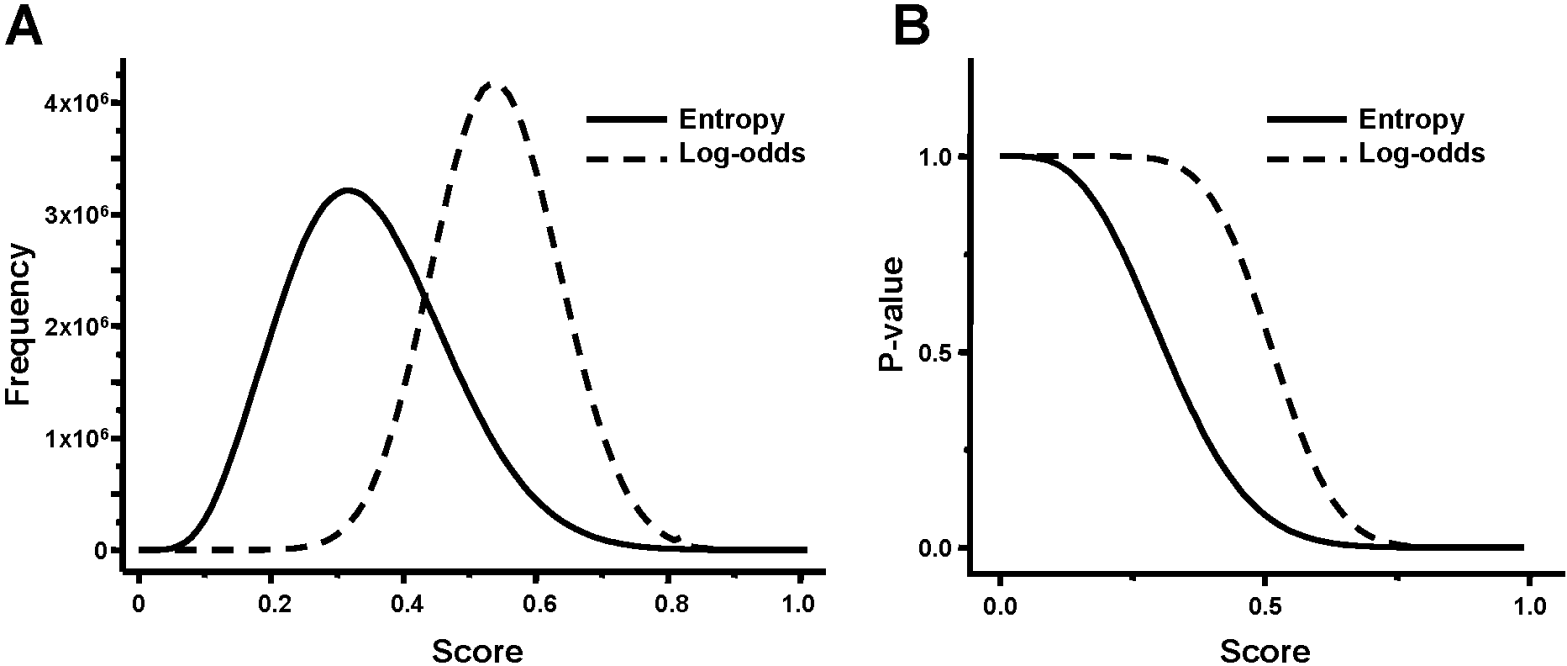
**Simulation-based P-value estimation**. A collection of 10^8 ^sites 23 nt-long were generated as third-order Markov chains using the *E. coli *nucleotide frequencies. The sites were then matched to the consensus shown in Table 1 with the entropy-weighted and log-odds algorithms. The respective score distribution are shown in Panel A. The P-values were estimated on the basis of the cumulated score distributions as shown in Panel B.

Since FITBAR allows two user-selectable DNA scanning algorithms (log-odds or entropy-weighted) and two user-selectable P-value algorithms (LMM or CIS), the four combinations were analyzed and the results are detailed in Table 2. It can be seen that globally, the entropy-weighted and log-odds screening methods yielded similar results even if the score values were more dispersed for the log-odds algorithm. A simulation was carried out to verify this behavior with a collection of 10^8 ^random sites modeled with third-order Markov chains. The distribution of log-odds scores was nearly symmetrical whereas the entropy-weighted distribution showed a positive skew (Figure 3A). The observed difference in score dispersion is therefore due to the PSSM scanning algorithms. Interestingly, the entropy-weighted scanning was able to find additional sites not detected by log-odds. They correspond to a strong site between *ddlA *and *iraP*, and weaker sites such as those upstream *tdk*, *hns*, *aer, patA *and others.

**Table 2:** Benchmarking results

#	Position	Gene(s)	Sequence	**Str**.	FITBAR (this work)								Simulation		**PRODORIC Virtual Footprint **[15]	**RSA Tools **[13]	**RegPredict **[16]
					
					Entropy-CIS		Entropy-LMM		Log odds-CIS		Log odds-LMM		Entropy	Log-odds	PWM Score	Score	Score
					Score	P-value	Score	P-value	Score	P-value	Score	P-value					
1	4538216	*nanC fimB*	cataattctcatcatgaaatatg	R	0.95026	0.01634	0.95026	4.6 E-09	0.81891	0.01012	0.81891	0.00006	2.0 E-07	0,00052	17.84	-	-
2	1819873	*chbB*	cttaattatcttcgcgaattatt	L	0.93979	0.01898	0.93979	7.5 E-09	0.83231	0.00710	0.83231	6.9 E-06	4.4 E-07	0,00035	17.59	1.0	5.58
3	707425	*chiP*	gataattcgcgtcgcgaaaaata	R	0.93979	0.01898	0.93979	2.8 E-08	0.71507	0.02168	-	-	4.4 E-07	0,01746	17.50	1.0	5.71
4	1899865	*yoaE manX*	gatattttacctttcgaaatttc	R	0.92147	0.02905	0.92147	1.1 E-07	0.80400	0.01257	0.80400	0.00008	1.3 E-06	0,00110	17.29	1.0	5.48
5	400463	*iraP ddlA*	aataattacccacacaaaatata	L	0.90052	0.04027	0.90052	8.1 E-07	-	-	-	-	3.5 E-06	-	-	-	-
6	1580605	*ydeN*	cttatttctcttcgtaaaattac	L	0.89921	0.04076	0.89921	6.6 E-07	0.84788	0.00414	0.84788	4.4 E-06	3.5 E-06	0,00015	16.85	1.0	5.53
7	3537939	*feoA*	ggtaattcactattcgaattata	R	0.89660	0.04179	0.89660	7.9 E-07	0.67381	0.03302	-	-	3.5 E-06	0,05546	16.85	-	5.15
8	703020	*nagB nagE*	tttaatttgcgatacgaattaaa	R	0.89660	0.04179	0.89660	4.9 E-07	0.69745	0.02567	0.69745	0.00198	3.5 E-06	0,02854	16.83	1.0	5.21
9	3086266	*galP*	cttaattcacaataaaaaataac	R	0.89267	0.04363	0.89267	5.7 E-07	0.72356	0.01813	0.72356	0.00121	3.5 E-06	0,01746	16.85	-	5.09
10	3718336	unknown	tttatttgttttcaggaaataaa	R	0.88482	0.05522	0.88482	1.9 E-06	-	-	-	-	5.6 E-06	-	-	-	-
11	703043	*nagE nagB*	tttaattcgtatcgcaaattaaa	L	0.87827	0.05835	0.87827	1.6 E-06	0.65996	0.03449	-	-	9.0 E-06	0,06789	-	-	5.15
12	3913456	*glmU*	gtttattcattgatcgaaataag	L	0.87304	0.06097	0.87304	1.4 E-06	0.68260	0.03175	0.68260	0.00097	1.4 E-05	0,04487	16.45	1.0	5.01
13	707448	*chiP*	tatttttcgcgacgcgaattatc	L	0.86780	0.06372	0.86780	5.6 E-06	-	-	-	-	1.4 E-05	-	-	-	-
14	1292271	*tdk hns*	atttattggcggcacaaaataaa	L	0.86649	0.06450	0.86649	5.2 E-06	-	-	-	-	1.4 E-05	-	-	-	-
15	2531523	*ptsH*	attattttgatgcgcgaaattaa	R	-	-	0.86387	3.2 E-06	-	-	-	-	2.1 E-05	-	-	-	-
16	3217267	*aer patA*	gttaattatcttgcccaaaaatc	R	-	-	0.86518	3.6 E-06	-	-	-	-	2.1 E-05	-	-	-	-
17	4633489	*rob creA*	gttatttaccgtgacgaactaat	R	-	-	0.86518	4.2 E-06	-	-	-	-	2.1 E-05	-	-	-	-
18	1120757	*dinI*	gttattttacctgtataaataac	L	-	-	0.86126	8.2 E-06	-	-	-	-	2.1 E-05	-	-	-	-
19	2573887	*eutS*	gttatttactctgacgaaaaatt	L	-	-	0.86126	8.7 E-06	-	-	-	-	2.1 E-05	-	-	-	-
20	3086289	*galP*	gttattttttattgtgaattaag	L	-	-	-	-	0.69755	0.02567	0.69755	0.00155	-	0,02854	-	-	-
21	702949	*nagE nagB*	cttattttatcattcaaaaaatc	L	-	-	-	-	0.68969	0.02876	0.68969	0.00095	-	0,03596	16.06	1.0	-
22	1580729	*ydeN*	gttgtttatcggcgagaaattac	L	-	-	-	-	0.65082	0.03846	-	-	-	0,08240	-	-	-
23	1584727	*ydeP*	cttattttttatattgaaaaata	L	-	-	-	-	-	-	-	-	-	-	16.21	1.0	4.82
24	2628932	*unknown*	gttttttatcttcaagaattata	L	-	-	-	-	-	-	-	-	-	-	16.11	-	-

				**Time (s)**	12.58 ± 1.13		10.58 ± 0.27		6.61 ± 0.34		6.14 ± 0.47		749.25 ± 12.22		5.10 ± 0.38	23.3 ± 1.21	4.6 ± 0.09

FITBAR was then compared to other available web servers such as RSA Tools [13], PRODORIC Virtual Footprint [15] and RegPredict [16]. The PredictRegulon web service was taken offline during this work and could not be tested. It appears that the results obtained by Virtual Footprint closely resemble FITBAR log-odds predictions (Table 2). Surprisingly, the highly ranking site located between *nanC *and *fimB *was not detected by RSA Tools and RegPredict. Another site, upstream *galP*, was detected by FITBAR, Virtual Footprint and RegPredict but was absent from RSA Tools predictions. Experimental data from several reports corroborate the regulatory role of these particular sites. First, repression of *nanC *and *fimB *divergent transcripts by NagC has been observed both *in vivo *and *in vitro *[32]. More recently, we were able to demonstrate by *in silico *genome screening followed by a combination of biochemical and genetic approaches that *galP *transcription is strongly repressed by NagC [33]; this finding provides a rationale for the better growth of *E. coli nagC *mutants on galactose [34]. These results show that the detection sensitivity of FITBAR equals or surpasses that of existing tools.

**Figure 4:**
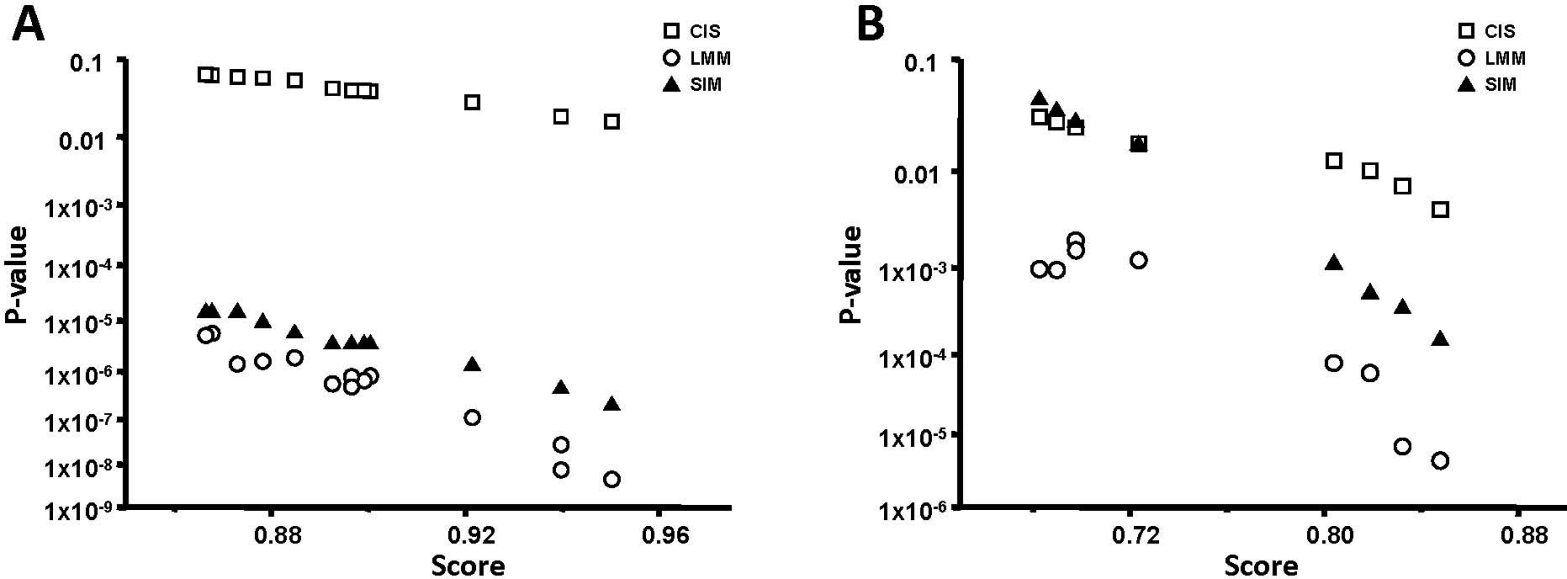
**Comparison between the simulated, LMM and CIS P-values as a function of the PSSM scores for the entropy-weighted (Panel A) or log-odds (Panel B) genome scanning algorithms**.

The use of two independent P-value algorithms, in combination with the biological data described above was instrumental in the reciprocal validation of the cutoff P-values. In the original descriptions of the CIS [21] and LLM [22] algorithms, the elimination of false positives is recommended for sites with a theoretical P-value above 10^-3 ^and 2 × 10^-4^, respectively. The benchmarking test shows that the LMM and CIS P-values differed significantly for each predicted site, up to several orders of magnitude (Table 2). Such variations in P-values for each predicted site were expected: they are due to the method used to model randomness in the P-value estimation procedures [17]. In this particular situation, they reflect presumably the impact of general versus local background distribution models. At this stage, it was important to compare the P-values obtained with CIS and LMM to those computed using a classical but slower method. Separate P-values were calculated for the 10^8 ^random samples in the above mentioned simulation using the log-odds and entropy-weighted scanning algorithms. Similarly to the PSSM scores, the P-values varied notably according to the genome scanning algorithm (Figure 3B). Simulated P-values could therefore be assigned to each predicted NagC operator in Table 2 to allow comparison between the different methods. Interestingly, all simulated entropy-weighted P-values were comprised between the corresponding LMM and CIS P-values and always within two orders of magnitude from LMM; on the other hand simulated and CIS P-values differed by five logs or less (Figure 4A). The difference between the simulated log-odds P-values and the LMM or CIS P-values never exceeded two orders of magnitude (Figure 4B). These results enabled the determination of the cutoff P-values to discriminate against false positives. In general, it was necessary to retain higher cutoff values than those recommended in the original CIS and LMM descriptions. The CIS cutoff P-values for the CIS method were set to 0.04 for the log-odds and 0.065 for the entropy-weighted algorithms. Corresponding P-values of 0.018 and 0.044 for the NagC operator upstream *galP *fit quite well within the cutoff values. A LMM cutoff P-value of 2 × 10^-3 ^was retained for the log-odds scanning method which accommodates the value for 0.0012 for *galP*. In the case of the entropy-weighted method, the observed LMM P-values after filtering were all lower than the recommended cutoff of 2 × 10^-4^. It would be interesting to analyze the biology of some of the weaker detected sites such as upstream *tdk *or *hns *to verify these findings.

The efficiency of FITBAR was compared to that of existing PSSM search web tools by measuring the respective response times from a network address outside the servers domains. The results indicated that FITBAR performance equals or exceeds to that of comparable tools and most importantly, that the calculation of the P-values does not impair significantly the general performance (Table 2). Finally, it is worth mentioning that the time required to calculate P-values by simulation exceeds by two to three orders of magnitude the time required by the CIS and LMM algorithms (Table 2).

## Conclusions

Current genomic TBFSs scanning programs do not provide P-values for the predicted sites and existing P-value computing algorithms have not been applied to the scanning of entire genomes in real time. In response to the gap in the available bioinformatics software, FITBAR was implemented as a performing workbench to assist experimentalists with the identification of regulons in prokaryotic genomes. The prediction of novel protein binding sites is achieved by a user-selectable combination of optimized sequence scanning and P-value calculation algorithms. In addition, this web tool presents a number of improvements. A rich user-friendly graphical interface presents a sequence logo for the query sites and precise genomic context map for each TFBS. The manual handling of large sequence files and cryptic parameter tweaking are eliminated. General performance equals or exceeds that of existing score-based PSSM scanning resources. Recently, FITBAR has been used to identify, in the *E. coli *genome, new high affinity targets for the N-acetylglucosamine repressor, NagC; the validity of the *in silico *predictions was confirmed by exhaustive genetic and biochemical evidence [33]. The effortless access to the prokaryotic genomes database, updated daily, permits the analysis of phylogenetically related organisms to validate regulon predictions. Finally, the annotation of new genomes and transcriptomic projects might benefit from this tool as well.

## Availability And Requirements

Project name: FITBAR

Project home page: http://archaea.u-psud.fr/fitbar

Operating system(s): platform independent

Programming language: C# and ASP.NET

Other requirements: Internet connection

License: none required

Any restrictions to use by non-academics: no restriction
